# Supplementary material for: Automated annotation of birdsong with a neural network that segments spectrograms
Source: eLife. 2022 Jan 20;11:e63853. doi: 10.7554/eLife.63853 (PMC8860439; doi:10.7554/eLife.63853)
Supplement: Transparent reporting form [file elife-63853-transrepform1.pdf]

eLIFE
1st Floor
24 Hills Road
Cambridge CB2 1JP, UK
P 01223 855340
W elifesciences.org
T @elife

eLife's transparent reporting form

We encourage authors to provide detailed information *within their submission* to facilitate the interpretation and replication of experiments. Authors can upload supporting documentation to indicate the use of appropriate reporting guidelines for health-related research (see EQUATOR Network), life science research (see the BioSharing Information Resource), or the ARRIVE guidelines for reporting work involving animal research. Where applicable, authors should refer to any relevant reporting standards documents in this form.

If you have any questions, please consult our Journal Policies and/or contact us: editorial@elifesciences.org.

Sample-size estimation

- You should state whether an appropriate sample size was computed when the study was being designed
- You should state the statistical method of sample size computation and any required assumptions
- If no explicit power analysis was used, you should describe how you decided what sample (replicate) size (number) to use

Please outline where this information can be found within the submission (e.g., sections or figure legends), or explain why this information doesn't apply to your submission:

We did not perform a power analysis. For our study of machine learning models, we did train multiple replicates on different randomly-drawn subsets of training data. We decided on the number of replicates based on good practices in machine learning, specifically the practice of performing *k*-fold cross validation, where *k* is typically set to 5-10 folds. As we specify in the methods, we trained 10 replicates for each dataset of an individual Bengalese finch's song (n=8 birds) and 7 replicates for each dataset of an individual canary's song (n=3 birds). This number of replicates was sufficient to demonstrate our training method and obtain population estimates of the metrics we used. Training a larger number of models would have been computationally expensive.

Because our method addresses a problem in an area where little work has been done, we compare our deep learning model with a different machine learning algorithm (Support Vector Machine). In figure 3 we use Wilcoxon's signed-rank test and Levene's test to compare the performance of models and its variance on a fixed test set across animals and training sets. We report p-values in the main text and provide details of data sets and numbers of replicates in the methods section "Use of existing datasets" and "Comparison with a Support Vector Machine model"

Replicates

- You should report how often each experiment was performed
- You should include a definition of biological versus technical replication
- The data obtained should be provided and sufficient information should be provided to indicate the number of independent biological and/or technical replicates
- If you encountered any outliers, you should describe how these were handled

- Criteria for exclusion/inclusion of data should be clearly stated
- High-throughput sequence data should be uploaded before submission, with a private link for reviewers provided (these are available from both GEO and ArrayExpress)

Please outline where this information can be found within the submission (e.g., sections or figure legends), or explain why this information doesn't apply to your submission:

This information does not apply to our submission because none of our studies involve biological replicates.

We do benchmark our machine learning algorithm by repeatedly training our model from randomly-initialized weights, and we do use the word "replicates" when clearly stating the number that we trained (e.g., the methods section "Comparison with a Support Vector Machine model" indicates 10 (7) replicates trained for each finch (canary) in the dataset). We chose these numbers of replicates based on the standard practice of *k*-fold validation, where *k* typically is 5-10 folds.

Statistical reporting

- Statistical analysis methods should be described and justified
- Raw data should be presented in figures whenever informative to do so (typically when N per group is less than 10)
- For each experiment, you should identify the statistical tests used, exact values of N, definitions of center, methods of multiple test correction, and dispersion and precision measures (e.g., mean, median, SD, SEM, confidence intervals; and, for the major substantive results, a measure of effect size (e.g., Pearson's r, Cohen's d)
- Report exact p-values wherever possible alongside the summary statistics and 95% confidence intervals. These should be reported for all key questions and not only when the p-value is less than 0.05.

Please outline where this information can be found within the submission (e.g., sections or figure legends), or explain why this information doesn't apply to your submission:

In the text describing results related to figure 3, we report statistical tests comparing model performance. In the figure itself we depict the mean and standard deviations, and report the number of animals and model training replicates in the legend. Since the statistical test is not a central point in our manuscript, we present the tests being carried and the p-values limiting a group of tests. We therefore do not include a specific value of all confidence intervals and effect sizes.

We use a method introduced by Warren et al. (2012) to compare transition probabilities in Bengalese finch 'branch points'. We reference that manuscript and provide a brief description in the methods section "Bengalese finch branch points". Since this test is used to show that probabilities do not change it is not appropriate to present (not significant) p-values. Instead, we write "Bonferroni-corrected pairwise bootstrap test, n.s." where appropriate.

The construction of probabilistic suffix trees (Fig. 7) involves a test referenced in the Methods sections:
"Probabilistic suffix trees"
and
"Model cross validation to determine minimal node frequency"

(For large datasets, or papers with a very large number of statistical tests, you may upload a single table file with tests, Ns, etc., with reference to sections in the manuscript.)

Group allocation

- Indicate how samples were allocated into experimental groups (in the case of clinical studies, please specify allocation to treatment method); if randomization was used, please also state if restricted randomization was applied
- Indicate if masking was used during group allocation, data collection and/or data analysis

Please outline where this information can be found within the submission (e.g., sections or figure legends), or explain why this information doesn't apply to your submission:

This information doesn't apply to our submission because none of the experiments include group allocation.

Additional data files ("source data")

- We encourage you to upload relevant additional data files, such as numerical data that are represented as a graph in a figure, or as a summary table
- Where provided, these should be in the most useful format, and they can be uploaded as "Source data" files linked to a main figure or table
- Include model definition files including the full list of parameters used
- Include code used for data analysis (e.g., R, MatLab)
- Avoid stating that data files are "available upon request"

Please indicate the figures or tables for which source data files have been provided:

Source data is provided for figures 3-6 and 8.
